# Using wastewater surveillance to investigate community-level differences in antibacterial resistance in a major urban center, USA

**DOI:** 10.1128/aem.01684-25

**Published:** 2025-10-30

**Authors:** Cameron Goetgeluck, Oluwatosin Olojo, Stephen P. Hilton, Orlando Sablon, Lorenzo Freeman, Patrick Person, David Clark, Robert H. Lyles, Timothy D. Read, Caroline Sheikhzadeh, Marlene K. Wolfe, Maya L. Nadimpalli

**Affiliations:** 1 Gangarosa Department of Environmental Health, Rollins School of Public Health, Emory University 1371, Atlanta, Georgia, USA; 2 Department of Epidemiology, Rollins School of Public Health, Emory University 1371, Atlanta, Georgia, USA; 3 Center for Global Safe WASH, Hubert Department of Global Health, Emory University 1371, Atlanta, Georgia, USA; 4 City of Atlanta Department of Watershed Management 711282 , Atlanta, Georgia, USA; 5 Fulton County Public Works, Atlanta, Georgia, USA; 6 Department of Biostatistics and Bioinformatics, Rollins School of Public Health, Emory University 1371, Atlanta, Georgia, USA; 7 Department of Medicine, Division of Infectious Diseases, Emory University School of Medicine 12239, Atlanta, Georgia, USA; Centers for Disease Control and Prevention, Atlanta, Georgia, USA

**Keywords:** wastewater surveillance, antimicrobial resistance, social determinants of health, antibiotic resistance gene

## Abstract

Wastewater surveillance could be a low-cost strategy for assessing patterns of antibiotic resistance across urban populations. We quantified fluoroquinolone-resistant (FQ-R) Enterobacterales, third-generation cephalosporin-resistant (3GC-R) Enterobacterales, carbapenem-resistant Enterobacterales, and the *bla*_CTX-M_-group 1 and *bla*_KPC_ genes in influent wastewater from 12 diverse sewersheds across metro Atlanta over two seasons; examined associations between culture- and digital PCR-based outcomes; and investigated relationships between sewersheds’ sociodemographic characteristics and concentrations of antibiotic-resistant bacteria in wastewater. FQ-R Enterobacterales, 3GC-R *Escherichia coli*, and 3GC-R *Klebsiella*, *Enterobacter*, or *Citrobacter* spp. (KEC) were detected in 79%–94% of samples (*n* = 34), with concentrations differing among sewersheds. Carbapenem-resistant *E. coli* and KEC were not reliably detected. The *bla*_CTX-M_-group 1 gene was detected in every sample, and we detected trends toward an association with 3GC-R *E. coli* and KEC, suggesting potential utility of this gene as a monitoring target. The *bla*_KPC_ gene was detected in every sample despite carbapenem-resistant *E. coli* and KEC not being reliably detected, suggesting it is not an appropriate indicator for these bacteria. After accounting for season, repeated measures, and potential healthcare inputs, we found that sewersheds with higher proportions of crowded households, Hispanic, non-Hispanic Asian, and individuals speaking a language other than English at home had higher flow-normalized concentrations of FQ-R Enterobacterales, 3GC-R *E. coli* and/or KEC in their wastewater. Given the potential for confounding, comparisons with human data are needed to determine how well sociodemographic patterns observed through wastewater mirror trends in the population.

**IMPORTANCE:**

Wastewater surveillance is a popular tool for the monitoring of health-related targets. Previous studies have demonstrated increases in antibiotic resistance among wastewater-derived fecal pathogens that match temporal trends in geographically-matched patient populations, indicating utility for assessing population-level patterns. Few studies have studied wastewater to examine antibiotic resistance patterns within a city or to identify sociodemographic characteristics associated with higher concentrations. We tested municipal wastewater from 12 sewersheds across metro Atlanta across two seasons. We identified significant differences in antibiotic-resistant bacterial concentrations across sewersheds, and after accounting for season, repeated sampling, and potential inputs from healthcare facilities, we found these differences were associated with community characteristics like living conditions and language. Overall, given that clinical surveillance is unlikely to be representative of the US population due to unequal healthcare access, wastewater surveillance merits consideration as a novel approach for antibiotic resistance surveillance.

## INTRODUCTION

Antibiotic resistance poses a significant and growing threat to global public health. Nearly 3 million Americans suffer from antibiotic-resistant (AR) infections annually (1). While antibiotic resistance has been a long-standing challenge in healthcare settings, resistance among community-acquired infections is increasing, which complicates empiric treatment.

Understanding community-level patterns of antibiotic resistance is critical for guiding appropriate antibiotic treatments and monitoring antibiotic resistance trends over time. In many American cities, community-level patterns are difficult to assess given the disjointed network of healthcare providers, urgent care centers, hospitals, and contract laboratories that support outpatient care. Some public health departments compile these data for outpatient prescribers (e.g., NYC Department of Health and Mental Hygiene) but the majority do not; in these cases, outpatient prescribers rely on hospital-based antibiograms which may overestimate resistance in the community (2). More broadly, because healthcare is not universal in the United States, using medical records to understand community-level antibiotic resistance patterns is inherently biased toward individuals who can access care. Finally, because healthcare data only capture active infections, the much larger fraction of persons who are gut-colonized with AR pathogens and who are, therefore, at higher risk for AR community-acquired infections (3, 4) are missed.

Wastewater surveillance could be an alternative strategy for assessing patterns of antibiotic resistance across urban populations. Regular monitoring of wastewater is becoming a standard tool for the surveillance of health-related targets (5). As an aggregate biological sample of entire communities, wastewater surveillance provides the opportunity to access information about health that is non-invasive, inexpensive, and unbiased by access to diagnostic testing resources. Importantly, wastewater can provide a composite biological sample of AR Enterobacteriales both colonizing and infecting the population, thereby providing insight that cannot be captured by any existing public health surveillance system in the United States. However, there are challenges with using wastewater to monitor population-level antibiotic resistance (6). Sewer microbiomes mostly consist of environmental-origin bacteria (e.g., *Arcobacter*, *Acinetobacter*) which can harbor and exchange clinically relevant ARGs with human pathogens (7). AR bacteria excreted by humans can also replicate in sewage conveyance systems and may persist as biofilms, which can decouple measurements of ARGs or AR bacteria at the WWTP level from population-level estimates. Nevertheless, several studies have demonstrated that increases in AR among wastewater-derived fecal pathogens over time match trends in geographically matched patient populations (8–10), supporting the use of wastewater surveillance to assess population-level trends.

Metro Atlanta is a highly socioeconomically diverse urban center in the United States and provides an ideal setting for exploring the feasibility of wastewater surveillance to identify community-level patterns in antibiotic resistance. Nearly 20% of Atlanta’s population lives in poverty (11), which is substantially higher the U.S. average in 2022 (12), and high rates of income inequality exist among its diverse population. Here, we sought to quantify AR bacteria in wastewater across greater Atlanta, focusing on phenotypes that complicate urinary tract infection (UTI) treatment (i.e., fluoroquinolone [FQ] and third-generation cephalosporin [3GC] resistance), as UTIs are most often caused by gut-origin bacteria likely to be excreted in wastewater. We also considered phenotypes that are of high public health relevance (i.e., carbapenem resistance). We compared culture- and digital PCR-based approaches to inform future surveillance studies and investigated potential relationships with community-level sociodemographic characteristics related to income, race, living conditions, and education.

## MATERIALS AND METHODS

### Study sites and sample collection

**Fig 1:**
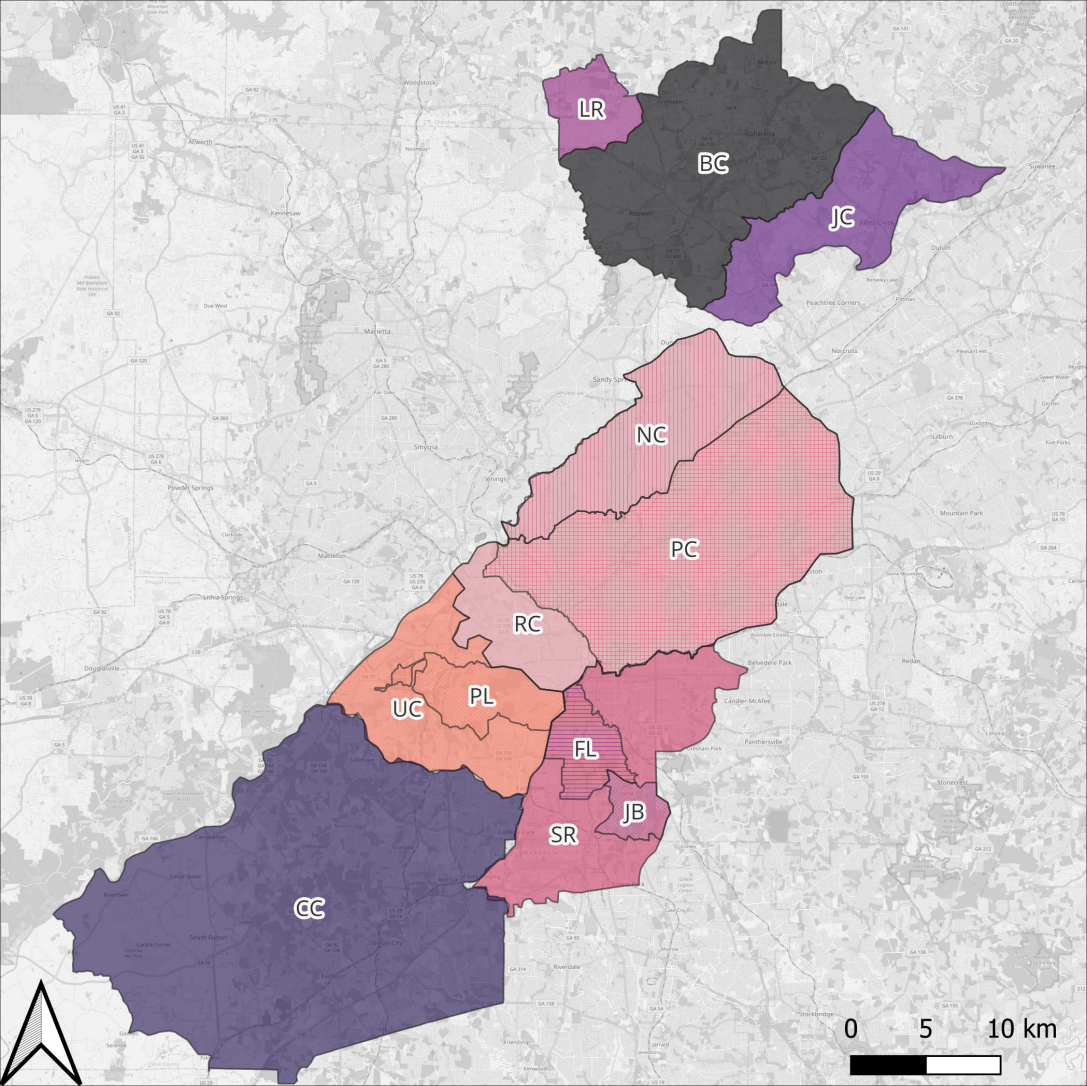
Map of sewersheds served by seven municipal wastewater treatment plants (WWTPs) and five influent lines sampled for this study. WWTPs include (starting at top, moving clockwise): LR = Little River, BC = Big Creek, JC = Johns Creek, RC = RM Clayton, SR = South River, CC = Camp Creek, and UC = Utoy Creek. Influent lines include two influent lines of RC, NC = Nancy Creek and PC = Peachtree Creek; two influent lines of SR, FL = Flint, JB = Jonesboro, and one influent lines of UC, PL = Phillip Lee. Atlanta sewershed shapefiles were obtained from the Atlanta Department of Watershed Management, and any areas outside city boundaries were estimated by digitizing maps from an online report (Dekalb County, 2022). Shapefiles for Fulton County were obtained from the Fulton County Department of Water Services. Background map data source: © OpenStreetMap contributors. Map prepared using QGIS 3.32 (QGIS Development Team, 2023).

Untreated influent wastewater was collected from seven municipal wastewater treatment plants (WWTPs) serving greater Atlanta and five influent lines feeding into three of these WWTPs (Fig. 1). We collected samples in December 2022, March 2023, and May 2023. WWTP-level samples were 24 h, composite, flow-proportional samples, while influent line samples were grab samples collected using 500 mL or 1 L Nalgene bottles. Wastewater samples were transported to the Rollins School of Public Health on ice and stored at 4°C until processing, typically within 1 h.

### Culture-based detection of antibiotic-resistant Enterobacterales

We aimed to isolate FQ-R, 3GC-R, and carbapenem-resistant Enterobacterales from wastewater using differential media. To select FQ-R Enterobacterales, we supplemented MacConkey agar with 4 µg/mL of ciprofloxacin (FQ plates). We used CHROMagar ESBL (CHROMagar Microbiology, Paris, France) to select 3GC-R *Escherichia coli* and KEC (*Klebsiella* spp., *Enterobacter* spp., and *Citrobacter* spp.) and CHROMagar mSuperCARBA (CHROMagar Microbiology, Paris, France) to select carbapenem-resistant *E. coli* and KEC.

Samples received in December 2022 were tested using membrane filtration. Briefly, wastewater was homogenized by shaking bottles laterally for 1 min. Fifty milliliters were centrifuged for 15 min at 4,000 rpm. We prepared 10-fold serial dilutions using phosphate-buffered saline (PBS). We used 10^−1^ through 10^−3^ dilutions to test for 3GC- and carbapanem-resistant Enterobacterales, while 10^−2^ through 10^−4^ dilutions were used for FQ-R Enterobacterales; pilot testing before study start revealed these dilutions to typically yield countable colonies (25–250 CFUs). Dilutions were vacuum filtered in duplicate through 0.45 µm membrane filters (Fisherbrand, Fisher Scientific, USA), which were aseptically plated onto FQ, mSuperCarba, and CHROMagar ESBL. Plates were incubated for 18 to 24 h at 37°C.

Given that we detected many of the outcomes of interest at high concentrations in December samples, we directly plated wastewater collected in March and May 2023. Briefly, untreated wastewater was left to settle at 4°C for approximately 45 min upon arrival in the lab. We prepared 1:5 and 1:10 dilutions in PBS and then plated 100 µL of each in duplicate on FQ, CHROMagar ESBL, and mSuperCarba plates. Plates were incubated for 18 to 24 h at 37°C.

We preferentially counted plates with 25–250 CFUs. Both tan (lactase non-producing) and pink (lactase-producing) colonies were counted on FQ plates. Pink (*E. coli*) and blue (KEC) colonies were counted on mSuperCarba and CHROMagar ESBL. For the FQ plates, our estimated lower limit of detection using either membrane filtration or a direct plating approach was 3 log_10_ CFU/100 mL. For mSuperCarba and CHROMagar ESBL, our estimated lower level of detection was 2 log_10_ CFU/100 mL when using membrane filtration and 3 log_10_ CFU/100 mL when directly plating.

### Species confirmation of cultured isolates

Up to three independent colonies of each desired phenotype were isolated and archived to confirm species using endpoint PCR. We used culture media that are selective and specific for Enterobacterales; however, these media (CHROMagar ESBL and mSuperCarba in particular) have largely been evaluated using human-origin samples. Because wastewater primarily comprises environmental-origin bacteria (7), we used PCR to confirm that colony appearance was still a reliable way to identify Enterobacterales species of interest. To confirm *E. coli* species, pink colonies from mSuperCarba and CHROMagar ESBL and both pink and tan colonies from FQ plates were screened for the *lacZ* and *yaiO* genes (13); isolates that harbored both genes were considered *E. coli*. All blue colonies from mSuperCarba and CHROMagar ESBL were tested for the presence of the *zikr* gene, which is specific to *K. pneumoniae* (14). PCR products were visualized using gel electrophoresis with 1.5% agarose gels stained with SYBR Safe.

We noted that no pink colonies on MSuperCarba were confirmed to be *E. coli,* and almost no blue colonies were confirmed to be *K*. pneumoniae (Table S1), the primary species of interest for this study. Given these results, findings from CHROMagar mSuperCARBA are not presented.

### Molecular detection of antibiotic resistance genes in wastewater

We used clinical surveillance data from the CDC’s Emerging Infections Program (EIP), in which Georgia participates, to inform our selection of ARG alleles to quantify. Specifically, EIP’s Multi-site Gram-negative Surveillance Initiative (MuGSI) conducts active population and lab-based surveillance for CRE and extended-spectrum beta lactamase-producing Enterobacterales (ESBL-E) to determine incidence, monitor temporal trends, identify risk factors for infection, and characterize dominant ARGs and strain types. EIP data indicate that 97% of 3GC-R infections in EIP catchment areas harbor an ESBL, most commonly encoded by *bla*_CTX-M-15_, which belongs to group 1 of *bla*_CTX-M_ variants (15). Site data from the Georgia EIP indicate that approximately 20% of carbapenem-resistant infections in greater Atlanta harbor a carbapenemase (Table S2), of which *bla*_KPC_ is the most common allele (16). Thus, we chose to measure *bla*_CTX-M_-group 1 and *bla*_KPC_ concentrations in wastewater. We did not measure ARGs conferring resistance to fluoroquinolone since high-level fluoroquinolone resistance (minimum inhibitory concentration >= 4 µg/L) is typically mediated through chromosomal mutations of the QRDR region, rather than ARGs (17).

We processed 9.6 mL of each wastewater sample to screen for *bla*_CTX-M_-group 1, *bla*_KPC_, and 16S rRNA gene. Each sample was supplemented with 150 µL each of CERES Nanotrap Microbiome A and B and 100 µL of CERES Nanotrap Enhancement Reagent 3 to enhance our capacity to concentrate and capture bacteria, following established protocols (APP-075). Supplemented samples were concentrated and extracted on the KingFisher Apex automated purification system (Thermo Scientific) utilizing the MagMAX Microbiome Ultra Nucleic Acid Isolation kit (Applied Biosystems). We prepared 1:10,000 dilutions of overnight cultures of AR-BANK#0109 and ATCC BAA-1705 to use as positive controls for the *bla*_CTX-M_-group 1 and *bla*_KPC_ genes, respectively; these were concentrated and extracted using the same procedures. The Nanotrap script for concentration & extraction using the KingFisher Apex was provided by CERES Nanosciences, Inc.

To quantify *bla*_CTX-M_-group 1, *bla*_KPC_, and the bacterial 16S rRNA gene in wastewater, a hydrolysis probe-based approach utilizing end-point PCR along with partitioning was performed using the Qiagen QIAcuity digital PCR platform. We used previously published primers and probe sequences (Table S3) and a ready to use QIAcuity Probe PCR kit. All reactions were run on a QIAcuity Four system using QIAcuity Nanoplate 26k 24-well plates with a QIAcuity Nanoplate Seal. No-template controls (NTCs) were included with every Qiagen QIAcuity 26k 24-well Nanoplate. NTCs consisted of Qiagen’s Probe PCR master mix and molecular grade water. For *bla*_CTX-M_-group 1 and *bla*_KPC_, we used 10 µL 4× Probe PCR Master Mix, 5 µL of enhanced GC, 3 µL of primer/probe mix, 12 µL of RNase-Free water, and 10 µL of diluted template DNA (1:10) for a total reaction volume of 40 µL per reaction per manufacturer’s recommendations. For 16S rRNA, 10 µL 4× Probe PCR Master Mix, 5 µL of enhanced GC, 2 µL of primer/probe mix, 13 µL of RNase-Free water, and 10 µL of diluted template DNA (1:10,000) were used for a total reaction volume of 40 µL per reaction. For both the *bla*_CTX-M_-group 1 and *bla*_KPC_ targets, final concentrations for forward and reverse primers were 600 nM, while the final concentration of probe was 300 nM. For the 16S rRNA target, the final concentration for forward and reverse primers was 400 nM, while the probe was 200 nM. The QIAcuity dPCR cycling parameters were based on the QIAcuity Probe PCR kit Quick-Start Protocol with modifications made to the 2-step cycling condition to fully optimize PCR conditions for each target. The dPCR cycling parameters for *bla*_CTX-M_-group 1 were 95°C for 2 min, followed by 40 cycles at 95°C for 30 s and 62°C for 15 s. For *bla*_KPC_ & 16S rRNA, dPCR cycling parameters were 95°C for 2 min, followed by 40 cycles at 95°C for 15 s and 57°C for 30 s. Analysis was conducted on the QIAcuity Software Suite (Qiagen, version 2.2.026) under 1D scatterplot for absolute quantification of ARGs. Common thresholds were set based on target fluorescence intensity between background/negative partitions and positive partitions relative to the positive control. We manually calculated concentrations of the 16s rRNA gene after subtracting out the number of positive partitions in the NTC (up to 6 of >23,000 valid partitions) (18).

### Characterization of the *bla*_CTX-M_ allele

Colonies originally isolated on CHROMagar ESBL that were confirmed to be either *E. coli* or *K. pneumoniae* were tested for the presence of the *bla*_CTX-M_ gene using methods previously described (6). We selected a random subset of up to 3 *bla*_CTX-M_-harboring *E. coli* and 3 *K*. *pneumoniae* per sewershed, per round, for sequencing. We sequenced the forward strand of the *bla*_CTX-M_ amplicon using Sanger sequencing and determined whether a *bla*_CTX-M_ allele belonging to group 1, 2, 8, 9, 25, 64, 151, or 137 was harbored using BLASTn. We used a 98% identity threshold to identify matches.

### Demarcation of sewersheds

Sewershed shapefiles for Atlanta WWTPs (RM Clayton [RC], South River [SR], and Utoy Creek [UC]) and two influent lines (Peachtreek Creek [PC] and Nancy Creek [NC], both of RC) were obtained from the Atlanta Department of Watershed Management, and shapefiles for Fulton County WWTPs (Little River [LR], Big Creek [BC], Johns Creek [JC] and Camp Creek [CC]) were obtained from the Fulton County Department of Water Resources. Shapefiles for Atlanta WWTP sewersheds only provided information within the city of Atlanta. The area covered by sewersheds that extended outside the city of Atlanta was estimated using manhole data from Fulton County and by digitizing maps from a Dekalb County report (19).

For influent lines for which shapefiles were not available (i.e., Flint [FL], Jonesboro [JB], and Phillip Lee [PL]), sewersheds were initially estimated using a network approach that identifies all manholes upstream of a collection point (20). This only estimated coverage within the city of Atlanta, which was refined using shapefiles from the Atlanta Department of Watershed Management, when available. Areas outside Atlanta were estimated by digitizing a map from a Dekalb County report (19).

### Identification of hospitals and long-term care facilities within sewersheds

We identified active hospitals (https://data.cms.gov/provider-data/dataset/xubh-q36u) and long-term care facilities (LTCFs) (https://data.cms.gov/provider-data/dataset/4pq5-n9py) registered with the Centers for Medicare & Medicaid Services (CMS) as of 2024. We tabulated the number of staffed beds in each facility using publicly available data from CMS or from the American Hospital Directory (https://www.ahd.com/). We identified facilities located within the boundaries of each sewershed using geocoded addresses. We used QGIS (https://www.qgis.org/) to evaluate our confidence in each assignment. High confidence assignments were squarely within the boundaries of a given sewershed or connected to sewer mains associated with certain sewersheds, while low confidence assignments were relatively close to sewershed boundaries. The number of tabulated hospital and LTCF beds per sewershed is reported in Fig. S1.

### Allocating census data to sewersheds

We used block group-level data from the US Census Bureau’s 2016–2020 American Community Survey (11) (released March 2022) to tabulate various sewershed-level characteristics of the catchment areas served by the WWTPs and influent lines we sampled. ACS data were downloaded using the TidyCensus package in R (21). Data from block groups allocated to a sewershed were aggregated to create summary statistics for each sewershed. When a block group was only partially within a sewershed, census variables were allocated to the sewershed using an area and population interpolation approach similar to Logan et al. 2014 (22). Briefly, the allocation ratio for the block group was estimated using the populations from blocks, which are smaller than block groups (23). When a block itself was only partially within a sewershed, census variables were allocated using the ratio of the area within the sewershed. This approach assumes that sociodemographic variables measured by the US Census are homogenous within a block.

The following characteristics were aggregated for each sewershed (Table S4): total population; median household income in the past 12 months (in 2021 inflation-adjusted dollars); percentage of population 5 years and over who speaks languages other than English at home; percentage of population having Medicaid/means-tested public coverage only; percentage of population who is uninsured; percentage of population over 18 for whom poverty status is determined and whose income in the past 12 months was below the poverty level; percentage of residents over 25 who have completed high school or an equivalent degree; percentage of population living in crowded conditions (i.e., >1.01 persons/room, not including bathrooms and kitchens); percentage of population who are Hispanic or Latino; percentage of population who are non-Hispanic White alone; percentage of population who are non-Hispanic Black alone; and percentage of population who are non-Hispanic Asian alone.

### Statistical analysis

Descriptive statistics were compiled to examine concentrations of culture-based outcomes and ARGs, overall and per round of sampling. We normalized concentrations of AR Enterobacterales, *bla*_CTX-M_-group 1, and *bla*_KPC_ detected in municipal WWTP wastewater by daily flow rates for each sampling date, as reported by City of Atlanta and Fulton County plant operators (Table S5). Daily flow rates approximate the total population contributing to a 24 h composite sample. Due to limitations in data availability, outcomes measured in influent line grab samples were normalized by average annual flow rates. Specifically, we report “flow-normalized concentrations” for each outcome that were calculated by multiplying concentrations of the outcome measured (i.e., either CFU/L or gene copies/L) by flow rate (L/day) (24). We additionally normalized ARG concentrations by concentrations of the bacterial 16S rRNA gene (ARG copies/16S rRNA copies).

We examined differences in the mean flow-normalized concentrations of each culture-based outcome (i.e., FQ-R Enterobacterales, 3GC-R *E. coli*, 3GC-RKEC) and each ARG (i.e., *bla*_CTX-M_-group 1, and *bla*_KPC_) between WWTP sewersheds and between rounds using a two-way analysis of variance (ANOVA) to assess main effects of these two factors. Post-hoc Tukey tests were conducted to compare means pairwise for each factor, controlling for multiple comparisons at an overall alpha level of 0.05. This modeling approach treats observations as independent, conditional on the sampling round and sewershed.

We examined whether concentrations of 3GC-R *E. coli* or KEC were associated with *bla*_CTX-M_-group 1 using a three-step modeling approach, accounting for repeated measures within each sewershed. Separate regressions were fit with 3GC-R *E. coli* and 3GC-R KEC as the outcome variables, treating *bla*_CTX-M_-group 1 as the predictor of interest. Indicator variables for the sampling rounds were included based on reference cell coding, together with their interactions with *bla*_CTX-M_-group 1 in order to investigate differences in the regression relationship across rounds. For each of the two models, we first tested the null hypothesis that the sampling round had no effect on the mean outcome (i.e., coincident regressions). If this hypothesis was rejected, we then tested the null hypothesis of parallel regressions, i.e., allowing the mean outcome to vary across sampling rounds but maintaining the same level of association with *bla*_CTX-M_-group 1 regardless of the round. In the models used to test for coincidence and parallelism, we utilized an unstructured error covariance matrix to account for correlations due to repeated measures. Subsequently, we used Akaike’s information criterion (AIC) (25) to select the error covariance structure for the final models, with the candidates including the unstructured, compound symmetric, independence, and diagonal but heteroscedastic structures. Based on the selected model (which exhibited parallelism in each case), we assessed the magnitude of association between the outcome and *bla*_CTX-M_-group 1, while also examining pairwise Bonferroni-corrected comparisons of model intercepts across rounds. As with the initial repeated measures ANOVA models looking at differences across sampling round, estimation was conducted via restricted maximum likelihood as implemented in the SAS MIXED procedure (26).

We examined whether the number of staffed hospital beds or certified LTCF beds in a sewershed were associated with differences in the flow-normalized concentrations of each culture-based outcome. To account for repeated measures from the same sewersheds, we used linear regression models with a generalized estimating equation (GEE) (27) and an unstructured correlation matrix, while controlling for sampling round. We selected an unstructured correlation structure using Quasi Information Criterion (QIC) implemented using the MESS package in R; compared to independence, ar(1), and exchangeable structures, models with unstructured correlation matrices typically had equivalent or lower QIC values. We performed a sensitivity analysis that excluded low confidence facility assignments; results were similar and not presented. Both the number of staffed hospital beds and the number of certified LTCF beds in a sewershed were significantly associated with concentrations of each culture-based outcome in a sewershed’s wastewater although effect sizes were uniformly near zero (Table S6). To build the most parsimonious model given our limited number of observations, we, thus, compiled the number of staffed hospital beds and the number of certified LTCF beds into a single variable for subsequent analyses.

Lastly, to evaluate associations between each sewershed-level characteristic (Table S4) and each of our culture-based outcomes of interest, we used linear regression models with a GEE and an independence correlation matrix to account for repeated measures from the same sewersheds, while controlling for sampling round and numbers of hospital and LTCF beds. We selected an independence covariance matrix after exploring QIC values for a subset of models using independence, unstructured, ar(1), and exchangeable structures; models with independence correlation matrices typically had equivalent or lower QIC values.

All analyses were conducted in RStudio (Version 2022.12.0+353) or SAS 9.4 (Cary, NC).

## RESULTS

### AR Enterobacterales and ARGs were frequently detected in municipal wastewater

A total of 34 samples was collected from seven WWTP and five influent lines over three sampling rounds (Table 1). Fluoroquinolone- and 3GC-R Enterobacterales were detected in the majority of wastewater samples (94.1% and 85.2%, respectively, Table S7). As noted above, given that most pink and blue colonies on MSuperCarba plates were not confirmed to be either *E. coli* nor *K. pneumoniae* (Table S2), concentrations of carbapenem-resistant *E. coli* and KEC are not reported. In support of a separate project, we performed shotgun metagenomic sequencing of all mSuperCarba growth from a wastewater sample collected in May 2023; most carbapenem-resistant organisms belonged to the families *Pseudomonadaceae*, *Moraxellaceae*, and *Aeromonadaceae* (Supplementary Material, Fig. S2), suggesting environmental rather than human origins.

**TABLE 1:** Summary of included samples^*a*^

Site	Type	No. of samples	Dates tested (month/day/year)
Big Creek	Treatment plant	3	12/12/2022, 03/28/2023, 05/22/2023
Camp Creek	Treatment plant	3	12/12/2022, 03/28/2023, 05/22/2023
Johns Creek	Treatment plant	3	12/12/2022, 03/28/2023, 05/22/2023
Little River	Treatment plant	3	12/12/2022, 03/28/2023, 05/22/2023
RM Clayton	Treatment plant	3	12/12/2022, 03/27/2023, 05/22/2023
South River	Treatment plant	3	12/12/2022, 03/28/2023, 05/22/2023
Utoy Creek	Treatment plant	3	12/12/2022, 03/27/2023, 05/22/2023
Flint	Influent line	2	12/12/2022, 05/22/2023
Jonesboro	Influent line	2	12/12/2022, 05/22/2023
Nancy Creek	Influent line	3	12/12/2022, 03/27/2023, 05/22/2023
Peachtree Creek	Influent line	3	12/12/2022, 03/28/2023, 05/22/2023
Phillip Lee	Influent line	3	12/12/2022, 03/27/2023, 05/22/2023

^*a*^ All samples were tested for the *bla*_KPC_ and *bla*_CTX-M-1_ group genes by digital PCR. All samples were tested for FQ-R Enterobacterales, 3GC-R *E. coli*, and 3GC-R KEC by plating on selective media. Samples collected on 12/12/2022 underwent membrane filtration prior to plating. All other samples were directly plated.

**Fig 2:**
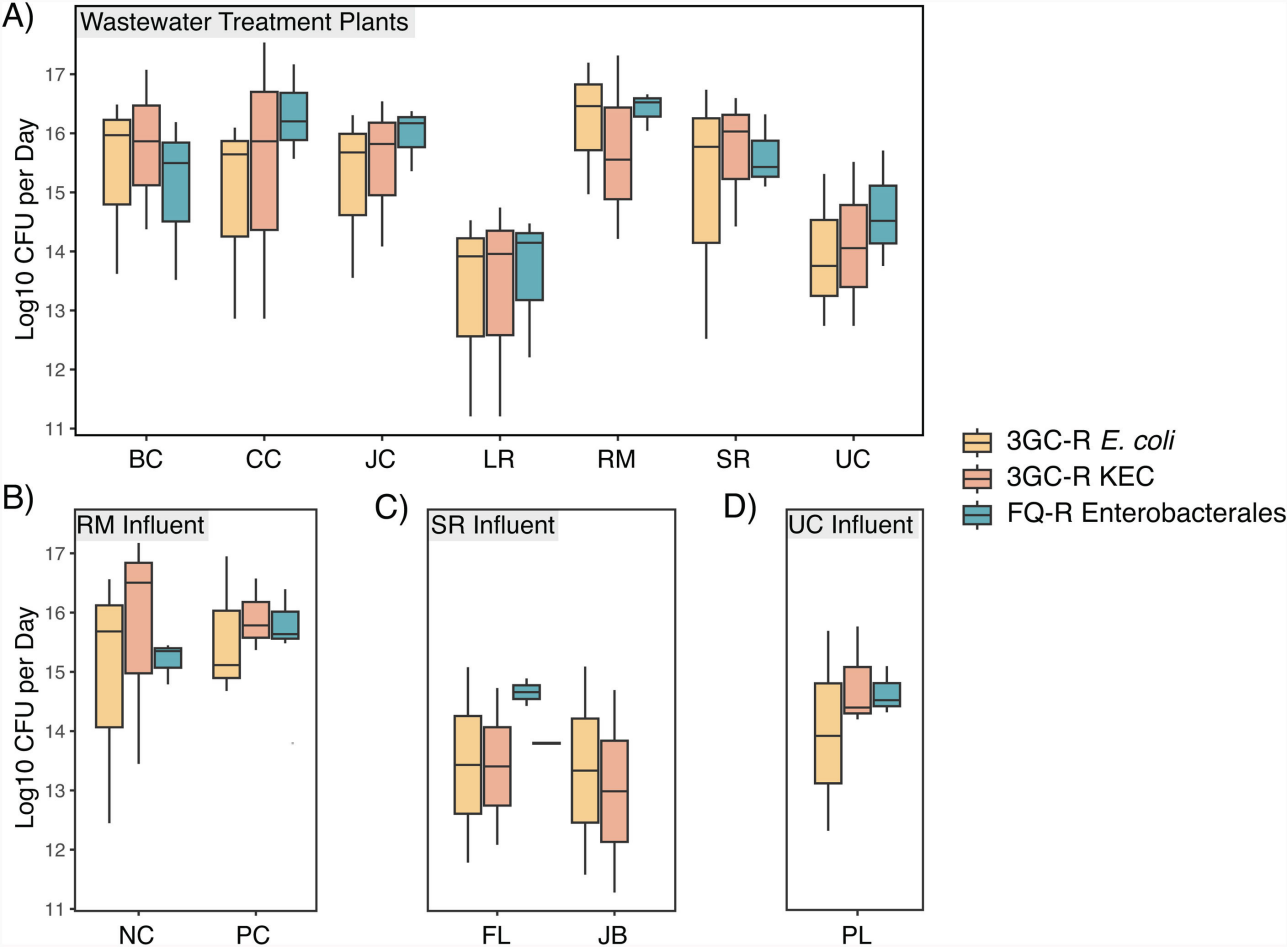
Concentrations of third-generation cephalosporin- and fluoroquinolone-resistant Enterobacterales in raw wastewater sampled in December 2022, March 2023, and May 2023 from (**A**) seven municipal wastewater treatment plants (WWTPs), (**B**) two influent lines of the RM Clayton WWTP, (**C**) two influent lines of the South River WWTP, and (**D**) one influent line of the Utoy Creek WWTP. Box plot whiskers depict the full range of concentrations observed, the bottom of each box depicts the 25th percentile, the horizontal line within each box depicts the median, and the top of each box depicts the 75th percentile. Concentrations for WWTPs have been normalized by average daily flow rates. Concentrations for influent lines have been normalized by average annual flow rates. *Note*: 3GC-R = third-generation cephalosporin-resistant and FQ-R = fluoroquinolone-resistant. BC = Big Creek, CC = Camp Creek, JC = Johns Creek, LR = Little River, RM = RM Clayton, SR = South River, UC = Utoy Creek, NC = Nancy Creek, PC = Peachtree Creek, FL = Flint, JB = Jonesboro, and PL = Phillip Lee.

Flow-normalized concentrations of 3GC-R *E. coli* and FQ-R Enterobacterales significantly differed across WWTP sewersheds after adjusting for multiple comparisons when accounting for sampling round (Fig. 2). Lower concentrations of both outcomes in Little River WWTP wastewater appeared to drive these global differences. Specifically, concentrations of 3GC-R *E. coli* were significantly lower in Little River WWTP sewage than in wastewater from the Johns Creek, Big Creek, South River, and RM Clayton WWTPs (by post-hoc Tukey Tests). Concentrations of 3GC-R *E. coli* were also lower in wastewater from the Utoy Creek WWTP compared to RM Clayton. Concentrations of FQ-R Enterobacterales were significantly lower in Little River WWTP sewage than in wastewater from the Johns Creek, Camp Creek, and RM Clayton WWTPs (by post-hoc Tukey Tests).

Average flow-normalized concentrations of 3GC-R *E. coli* and 3GC-R KEC increased between December and March sampling rounds (by post-hoc Tukey tests) but not between March and May, potentially reflecting seasonal differences between winter and spring (Fig. S3) or differences in laboratory testing methods (membrane filtration vs direct plating). There was no significant difference in average flow-normalized concentrations of FQ-R Enterobacterales across rounds after accounting for sewershed effects (*P* = 0.07 by two-way ANOVA). We observed greater variability in concentrations of 3GC-R *E. coli* and KEC relative to FQ-R Enterobacterales, potentially due to technical challenges in accurately counting pink and blue colonies given substantial overgrowth of other colony morphologies, as others have noted (28), or this could reflect actual variability in wastewater across sampling points.

**Fig 3:**
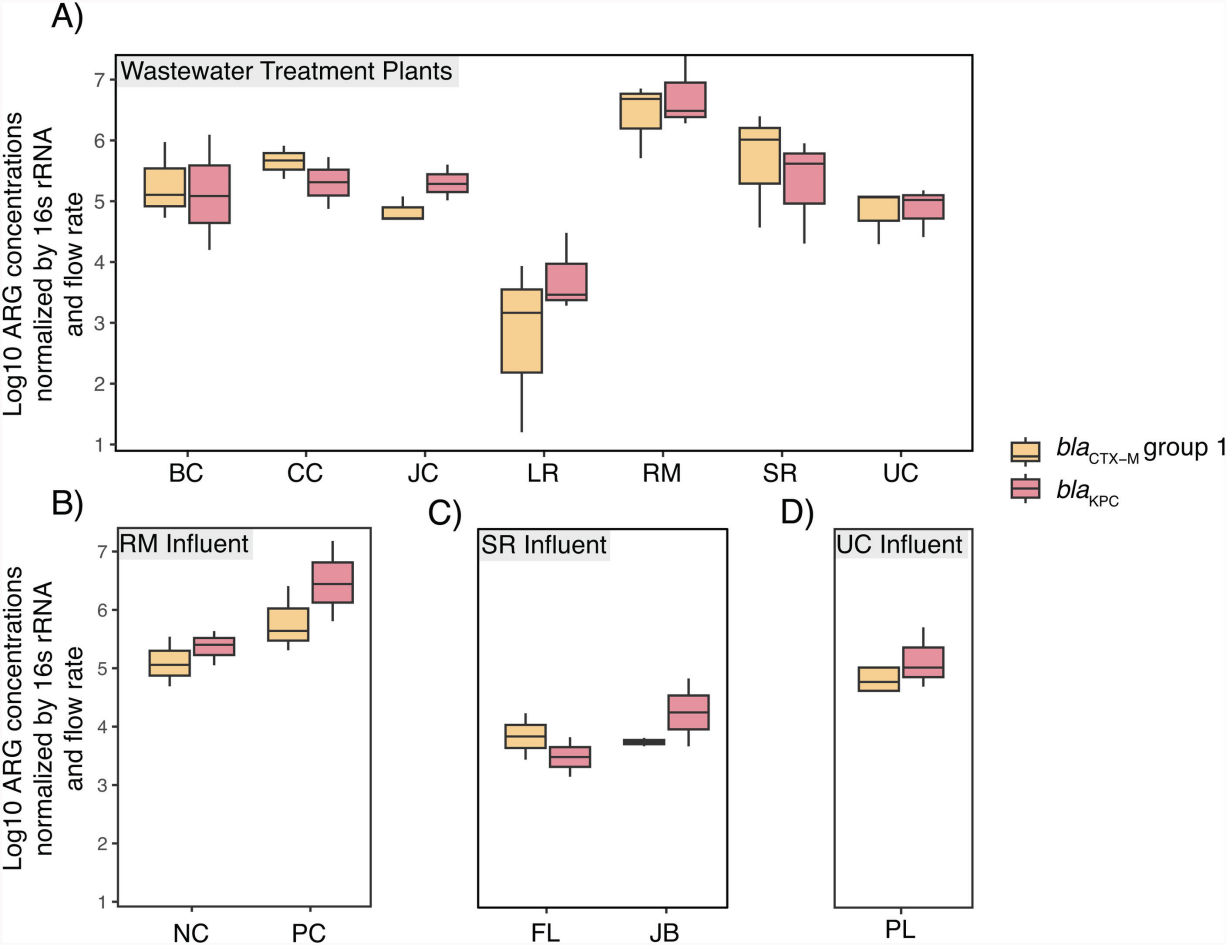
Concentrations of the *bla*_CTX-M_-group 1 and *bla*_KPC_ genes in raw wastewater sampled in December 2022, March 2023, and May 2023 from (**A**) seven municipal wastewater treatment plants (WWTPs), (**B**) two influent lines of the RM Clayton WWTP, (**C**) two influent lines of the South River WWTP, and (**D**) one influent line of the Utoy Creek WWTP. ARG concentrations were normalized by copies of the 16S rRNA gene prior to log transformation. Box plot whiskers depict the full range of concentrations observed, the bottom of each box depicts the 25th percentile, the horizontal line within each box depicts the median, and the top of each box depicts the 75th percentile. *Note*: For WWTPs, ARG concentrations were normalized by 16S rRNA concentrations and average daily flow rates. For influent lines, ARG concentrations were normalized by 16S rRNA concentrations and average annual flow rates.

We detected *bla*_CTX-M_-group 1 and *bla*_KPC_ in all samples with 16S rRNA normalized concentrations ranging from 4.8 × 10^−6^ to 0.01 and 1 × 10^−4^ to 0.05, respectively (Table S8). Flow-normalized concentrations of *bla*_CTX-M_-group 1 and *bla*_KPC_ significantly differed across WWTP sewersheds after adjusting for multiple comparisons when accounting for sampling round (Fig. 3). Again, significantly lower concentrations of both ARGs in Little River WWTP wastewater relative to other WWTPs (i.e., RM Clayton, Big Creek, Camp Creek, Johns Creek, South River, and Utoy Creek for *bla*_CTX-M_-group 1; RM Clayton, Camp Creek, Big Creek, Johns Creek, South River, and Utoy Creek for *bla*_KPC_) largely drove these global results although pairwise differences between other WWTPs were noted. Concentrations of *bla*_CTX-M_-group 1 were higher in March than in December, but there was no statistical difference in concentrations between March and May (by post-hoc Tukey tests); mirroring patterns observed for 3GC-R *E. coli* and 3GC-R KEC. In contrast, concentrations of *bla*_KPC_ did not differ between December and March but were higher in May than in March (by post-hoc Tukey tests) (Fig. S3).

### Correlations between AR Enterobacterales and ARGs were not consistent

We examined whether *bla*_CTX-M_-group 1 was associated with concentrations of 3GC-R *E. coli* or KEC to gain insight into the interpretation of *bla*_CTX-M_-group 1 measurements in wastewater. For both outcomes, the association with *bla*_CTX-M_-group 1 was found to be homogeneous across sampling rounds; that is, a repeated measures regression model assuming parallelism was selected. Thus, we applied linear mixed effects models assuming parallelism and using a diagonal error covariance matrix to evaluate each association. Table 2 displays the key results of the final selected models, including which error covariance structure was selected via AIC. Although neither relationship was statistically significant at the 0.05 level, we identified trends toward a positive association between *bla*_CTX-M_-group 1 and both 3GC-R *E. coli* (Estimate = 0.37, SE = 0.21, Pr > |*t*| = 0.10) and 3GC-R KEC (Estimate = 0.45, SE = 0.26, Pr > |*t*| = 0.10).

**TABLE 2:** Associations between concentrations of third-generation cephalosporin-resistant (3GC-R) *E. coli* and 3GC-R KEC in wastewater versus concentrations of the *bla*_CTX-M_-group 1 gene^*a*^

Outcome	Estimate	Std. error	Pr > |*t*|	Covariance structure^*b*^
3GC-R *E. coli*	0.370	0.212	0.097	Independent
3GC-R KEC	0.447	0.261	0.103	Diagonal

^*a*^ 3GC-R = third-generation cephalosporin-resistant.

^*b*^ Akaike’s information criterion (AIC) used to select error covariance structure for each final model. Candidates included unstructured, compound symmetric, independence, and diagonal but heteroscedastic structures.

### Distributions of *bla*_CTX-M_ alleles among *3GC-R E. coli* and *K. pneumoniae* were similar across sewersheds

More than 70% of tested pink colonies from CHROMagar ESBL were confirmed to be *E. coli*, and approximately 25% of tested blue colonies from CHROMagar KEC were confirmed to be *K. pneumoniae* (Table S1). We successfully sequenced the *bla*_CTX-M_ amplicon for 41 3GC-R *E. coli* and 24 3GC-R *K. pneumoniae*. The vast majority of *E. coli* and all *K. pneumoniae* harbored *bla*_CTX-M_-group 1 alleles although *bla*_CTX-M_-group 9 alleles were also detected among *E. coli* (Table S9; Fig. S4). There were no differences in the proportion of sequenced *E. coli* isolates encoding *bla*_CTX-M_-group 9 across sewersheds (*P* = 0.22 using a 12-sample test for equality of proportions without continuity of correction).

### Sociodemographic characteristics were associated with the detection of AR Enterobacterales in wastewater

Sociodemographic characteristics varied widely across sewersheds (Table 3). Specifically, we noted sizeable differences in median household income (min = $31,927, max = $173,500), proportion of residents lacking health insurance (min = 4.8%, max = 20.0%), and racial and ethnic makeup (e.g., 90.3% non-Hispanic Black in Utoy Creek sewershed versus 7.3% in Little River sewershed).

**TABLE 3:** Average sociodemographic characteristics of included sewersheds^*a*^

	Big Creek	Camp Creek	Johns Creek	Little River	RM Clayton	South River	Utoy Creek	Nancy Creek^*b*^	Peachtree Creek^*b*^	Flint^*c*^	Jonesboro^*c*^	Phillip Lee^*d*^
Type	WWTP	WWTP	WWTP	WWTP	WWTP	WWTP	WWTP	Influent line	Influent line	Influent line	Influent line	Influent line
Total population	191,555	182,543	85,119	14,308	588,510	149,274	97,144	123,159	407,917	31,219	3,744	35,756
Median household income (2020 inflation adjusted $)	115,797	49,679	134,285	173,500	86,971	52,288	36,860	114,688	87,704	31,927	51,987	40,881
% with public insurance	4.82	17.22	3.63	1.39	7.13	18.55	25.04	4.09	6.62	29.04	24.24	20.14
% uninsured	9.33	11.12	6.00	4.75	10.63	14.57	15.80	8.24	10.89	16.84	20.04	15.36
% below poverty line	4.54	10.26	3.72	2.72	11.11	18.94	18.87	6.24	10.62	27.06	28.00	16.11
% living in crowded households	2.33	3.00	2.42	0.23	2.12	1.93	2.00	1.70	2.25	2.26	0.23	0.52
% speaks a language other than English at home	29.00	7.43	33.14	5.62	21.84	9.98	4.11	23.11	23.64	7.45	3.34	2.90
% completed at least high school	95.18	91.79	96.32	97.66	92.60	87.35	88.53	96.01	92.66	83.59	88.65	86.54
Race/ethnicity (%)												
Hispanic or Latino	12.21	3.91	6.45	5.52	11.69	7.20	2.78	10.67	13.06	3.83	2.42	2.22
Non-Hispanic White	55.10	6.56	56.77	79.24	52.00	26.22	4.48	62.10	54.52	8.49	2.91	3.24
Non-Hispanic Black	14.28	87.28	9.63	7.28	24.03	60.68	90.35	13.20	19.70	83.65	90.45	91.30
Asian	13.42	0.56	23.54	3.01	9.00	2.11	0.28	11.59	9.11	1.34	0.01	0.26

^*a*^ Data from block groups allocated to a sewershed were aggregated to calculate means for each sewershed. When a block group was only partially within a sewershed, census variables were allocated to the sewershed using an area and population interpolation approach similar to Logan et al. 2014 (22). Briefly, the allocation ratio for the block group was estimated using the populations from blocks, which are smaller than block groups (23). When a block itself was only partially within a sewershed, census variables were allocated using the ratio of the area within the sewershed. This approach assumes that sociodemographic variables measured by the UC Census are homogenous within a block.

^*b*^ Influent line of RM Clayton WWTP.

^*c*^ Influent line of South River WWTP.

^*d*^ Influent line of Utoy Creek WWTP.

We identified four sewershed-level characteristics that were associated with flow-normalized AR Enterobacterales concentrations in untreated influent wastewater when accounting for repeated measures from sewersheds, sampling round, and the number of hospital or LTCF beds within each sewershed, including percent of persons living in crowded households, percent identifying as Hispanic or Latino, percent identifying as non-Hispanic Asian only, and percent speaking another language at home (Table 4). We note that the same sewershed-level characteristics were found to be significantly associated with concentrations of AR Enterobacterales that were not corrected for flow rate, indicating that these findings were not driven by differences in flow rate across sewersheds alone (Table S10). Results from all models are reported in Table S11.

**TABLE 4:** Statistically significant associations between flow-normalized concentrations of third generation cephalosporin-resistant (3GC-R) *E. coli*, 3GC-R KEC, and fluoroquinolone-resistant (FQ-R) Enterobacterales in Atlanta wastewater and sewershed-level sociodemographic characteristics, 2022–2023^*a*^

Sewershed-level characteristic	Outcome (log_10_ CFU/day)	Estimate	Std. error	Wald test statistic	Pr (>|*W*|)
% Living in crowded households^*b*^	3GC-R *E. coli*	0.50	0.12	16.88	3.98E-05
	3GC-R KEC	0.61	0.19	10.24	1.38E-03
	FQ-R Enterobacterales	0.70	0.14	26.43	2.73E-07
% Hispanic^*c*^	3GC-R *E. coli*	0.12	0.03	17.75	2.51E-05
	3GC-R KEC	0.17	0.04	19.66	9.24E-06
% Non-Hispanic Asian^*d*^	3GC-R *E. coli*	0.06	0.01	17.27	3.23E-05
	3GC-R KEC	0.06	0.02	8.51	3.53E-03
% Speaks language other than English at home^*e*^	3GC-R *E. coli*	0.05	0.01	34.30	4.73E-09
	3GC-R KEC	0.05	0.01	15.83	6.91E-05

^*a*^ Models accounted for repeated measures within each sewershed, sampling round, and number of hospital or long-term care facility beds within each sewershed. Statistical significance was defined as *P* < 0.167. Results from all models, regardless of statistical significance, are reported in Table S11.

^*b*^ Defined as greater than 1.01 persons/room, not including kitchens or bathrooms. Tabulated as number of owner-occupied households with greater than 1.01 occupants/room divided by total households.

^*c*^ Tabulated as number of persons self-identifying as Hispanic or Latino divided by the total population.

^*d*^ Tabulated as number of persons self-identifying as non-Hispanic Asian alone divided by the total population.

^*e*^ Tabulated as the number of persons 5 years and over who speak a language other than English at home divided by the total population. This variable was highly correlated with percent Hispanic or Latino (*ρ* = 0.80) and percent non-Hispanic Asian living in a sewershed (*ρ* = 0.95).

Percent of persons living in crowded households was significantly associated with higher concentrations of all three culture-based outcomes of interest we examined, i.e., FQ-R Enterobacterales (*β*: 0.70, SE: 0.14, *P*-value < 0.001), 3GC-R *E. coli* (*β*: 0.50, SE: 0.12, *P*-value < 0.001), and 3GC-R KEC (*β*: 0.61, SE: 0.19, *P*-value = 0.001). Household crowding was not strongly correlated with any of the other sociodemographic characteristics we evaluated per sewershed (*ρ* < 0.7 for all pairwise comparisons, Table S12) although a moderate correlation with percent speaking a language other than English at home was noted (*ρ* = 0.5).

Percent Hispanic or Latino, percent non-Hispanic Asian, and percent speaking a language other than English at home were all associated with higher concentrations of 3GC-R *E. coli* and 3GC-R KEC in a sewershed’s wastewater (Table 3) although effect sizes were small. Percent speaking a non-English language at home was strongly correlated with percent Hispanic or Latino (*ρ* = 0.80, Table S12) and percent non-Hispanic Asian (*ρ* = 0.95, Table S12) at a sewershed-level, and percent Hispanic or Latino and percent non-Hispanic Asian were moderately correlated with each other (*ρ* = 0.6). Sociodemographic characteristics related to income, insurance status, and higher education status were not strongly correlated with any of these variables at the sewershed level (*ρ* < 0.7 for all).

## DISCUSSION

Antibiotic-resistant bacteria of clinical concern are readily detected in metro Atlanta wastewater. We identified differences in the concentrations of 3GC-R *E. coli,* 3GC-R KEC, and FQ-R Enterobacterales in influent wastewater from 12 diverse sewersheds across Atlanta. Our analysis suggests that these differences may be at least partly explained by sociodemographic characteristics of these sewersheds, including living conditions, language spoken at home, and race and ethnicity. Additionally, our findings suggest that the *bla*_CTX-M_-group 1 gene could be an appropriate surrogate for third generation cephalosporin resistance among human fecal pathogens in wastewater, at least in this setting, which supports the use of this gene in wastewater surveillance. Overall, this study indicates that wastewater surveillance could provide new insight into community-level patterns of AR bacteria in urban centers in the United States though additional work is needed to validate how well these data approximate patterns of human colonization and infection with these bacteria.

Relatively few studies have examined community-level differences in antibiotic-resistant bacteria or ARGs in wastewater from urban centers (24, 29–31). One 2009 study in New York City reported concentrations of seven ARGs over one year across the five boroughs (Manhattan, Queens, Brooklyn, the Bronx, and Staten Island) (31). Although some differences between boroughs were noted, they were not further explored. At a national scale, a recent preprint by Kim et al. examined concentrations of 11 ARGs in wastewater solids from 163 sewersheds across the United States, and reported several associations with sewersheds’ sociodemographic characteristics, including proportion speaking English and housing conditions (32). International studies have also examined differences in AR bacteria and ARGs in wastewater from distinct neighborhoods (24, 29, 30, 33). Among three “socio-spatially” distinct districts of one city in western Germany, a 2021 study (29) reported consistently higher concentrations of ESBL-producing *E. coli* in wastewater from the most disadvantaged community over 1 year. Because the authors’ definition of disadvantage was not clearly defined, however, it is unclear whether some of the notable characteristics we identified in this study—for example, household crowding—may have been more common in the community they identified to be at higher risk. A study of 25 municipal WWTPs in greater Sydney, Australia, found that ESBL-producing Enterobacterales were commonly detected in influent and that differences in concentrations between catchments were significantly associated with the proportion of a catchment’s population that was between 19 and 50 years old and had completed vocational education (24). Two studies in Basel and Copenhagen also examined AR bacteria or ARGs in influent wastewater from distinct neighborhoods. Differences by neighborhoods’ sociodemographic characteristics were not statistically significant in Basel (30) and not investigated in Copenhagen (though study authors acknowledged limited variation in healthcare access, sanitation, income, ethnicity, and demographics across the sampled neighborhoods, which could have precluded such analyses) (33). To our knowledge, this is one of the first US studies to report specific sociodemographic characteristics that are associated with higher concentrations of clinically relevant AR bacteria in wastewater. The biological mechanisms underpinning the associations we observed remain unclear and could be due to differences in healthcare access (34), community antibiotic use (35, 36), diet (37), recent travel or immigration (38–40), or other factors that we were unable to account for. However, our findings are concordant with medical record studies that have repeatedly identified area-level characteristics as risk factors for patients’ colonization or infection with AR pathogens, including area-level income (41, 42), ethnic composition (34), insurance access (34), deprivation (43), and household living conditions (44), among others (45). Nevertheless, given the potential for substantial confounding (including from differences in sewer infrastructure), comparisons to human colonization and infection data are needed before wastewater data are leveraged to inform antibiotic resistance surveillance activities. 

Among the statistically significant sociodemographic characteristics we identified, crowding had the largest effect size. Crowding is a well-known risk factor for the spread of infectious diseases like methicillin-resistant *Staphylococcus aureus* and has been associated with higher risks for colonization and infection with other pathogens (45). The proportion of crowded households in Atlanta sewersheds ranged from 0.2% to 3.5%. Here, we found that a 1% increase in the number of crowded households in a sewershed was associated with three to five times higher concentrations of 3GC-R *E coli,* 3GC-R KEC, or FQ-R Enterobacterales per day in Atlanta wastewater. Interestingly, household crowding was not strongly correlated with any other sewershed level characteristics that we considered, including poverty or income. The US Department of Housing and Urban Development defines crowding as >1.01 persons/room, excluding bathrooms and kitchens. Tolerance or preference for living in multigenerational or multifamily housing that might be considered “crowded” by HUD’s definition can vary according to cultural background and may occur irrespective of a household’s income status. Given that there is no clear evidence that crowding is associated with higher rates of antibiotic consumption, the underlying drivers of the associations we observed merit further study.

Although we consistently detected the *bla*_KPC_ gene in wastewater, we were unable to reliably culture carbapenem-resistant *E. coli* or KEC from matched samples. Other US studies have also noted challenges in isolating carbapenem-resistant *E. coli* and KEC for species confirmation from wastewater (46). The majority of carbapenem-resistant bacteria in wastewater appear to belong to the families *Aeromonadaceae* (47, 48) and *Pseudomonadaceae*, which can grow on chromogenic media meant to be selective and differential for Enterobacterales (48), thereby severely complicating our ability to reliably detect carbapenem-resistant *E. coli* or KEC by culture. Carbapenem-resistant Enterobacterales infections are relatively rare in metro Atlanta and only a fraction (9%–25%) harbor any carbapenemase gene (Table S2). Our detection of low concentrations of the *bla*_KPC_ gene in every sample, therefore, was unusual. Overall, this suggests that the *bla*_KPC_ gene is likely commonly harbored by environmental bacteria and, thus, may not be an appropriate surrogate for carbapenem-resistant human fecal pathogens in municipal wastewater. We note that this has implications for the US CDC’s plans to consider implementing *bla*_KPC_ monitoring in US wastewaters; specifically, the public health interpretations of this target are unclear.

We accounted for the number of hospital beds and LTCF beds in a sewershed when assessing associations between sociodemographic characteristics and concentrations of AR bacteria in wastewater, but, like others (49, 50), we found their effects to be near negligible. Given the high volume of antibiotics used in hospital and LTCF settings, both are thought to be important point sources for AR bacteria and ARGs to municipal wastewater. While some studies have found that the presence of hospitals in a sewershed is associated with higher concentrations of AR bacteria in municipal WWTP influent (30), others have found that any signal from hospitals appears to “wash out” by the time of arrival at a municipal WWTP (49, 50). Whether or not hospitals and LTCF meaningfully contribute to the concentrations of AR bacteria in municipal WWTP wastewater may depend on per capita outpatient antibiotic consumption in a given community; healthcare inputs may be more likely to have negligible effects in settings where community antibiotic consumption is relatively high.

Further work is needed to determine whether the sewershed-level differences we observed in wastewater are reflective of population-level patterns of colonization or infection with AR bacteria. This is challenging for several reasons. First, the majority of AR human fecal bacteria excreted in wastewater are likely commensal organisms. However, data from non-healthcare-associated populations on the frequency of colonization with AR pathogens are rare in the United States, which limits the ability to perform such comparisons. Second, fecal bacteria like *E. coli* and *K. pneumoniae*, which were of particular interest in this study, may also originate from non-human sources including domestic pets (51), wild animals (52), and food waste (53) although the relative importance of these inputs is unclear in Atlanta. Livestock may also be a major contributor of AR fecal bacteria in other settings although no animal farms, slaughtering plants, or meat processing plants are permitted to discharge waste into the sewersheds we sampled. Third, AR bacteria could also replicate in sewer systems or previously susceptible fecal pathogens could acquire ARGs through horizontal transfer, particularly if bioavailable antibiotics, heavy metals, or other compounds exerting selection pressure are cooccurring in the system, although there is little experimental evidence for this (54). Despite these challenges, multiple studies have identified clinically relevant AR strains in wastewater (32), while others have demonstrated increases in AR among wastewater-derived fecal pathogens over time that match trends in geographically matched patient populations (8–10). Overall, this suggests that wastewater may provide high-level, “average” information about patterns of antibiotic resistance in sewershed populations, even if exact population estimates of AR burden are challenging to calculate given the reasons outlined above. We note that any insight gleaned by wastewater surveillance could be especially valuable in metro Atlanta where one in five residents lack health insurance and, therefore, is unlikely to be captured in any clinical surveillance.

Although community-level antibiotic consumption is likely an important driver of the differences we observed in AR bacteria concentrations across sewersheds (29, 55), we were not able to investigate those differences here. Existing databases (i.e., IQVIA, Epic Cosmos) report data at the county or ZIP-code level, which are not granular enough for gauging city-level differences, and further do not capture non-prescription use, which has been reported among 5% of US residents with higher rates in some communities (35, 56). A recent preprint reporting ARG concentrations in wastewater solids from 163 WWTPs across the US found that sewershed-level antibiotic prescribing (as tabulated using Epic Cosmos data) was either not correlated or only weakly positively correlated with ARG targets (Spearman correlation coefficient < 0.27 for all) (32). The degree to which non-prescription antibiotic use in US communities may confound associations between sewersheds’ sociodemographic characteristics and AR bacteria concentrations in wastewater is not known and is a limitation of this and similar studies.

Surveying municipal wastewater for AR bacteria to make population-level inferences poses several methodological challenges, which we attempted to mitigate or account for here. First, AR bacterial concentrations in wastewater may fluctuate during the day, reflecting changes in the sewershed population’s sewer use (57). We accounted for these fluctuations by collecting 24 h, composite flow-proportional samples from WWTPs. However, only grab samples were available from influent lines which may not be representative. Second, using WWTP-level measurements to make population-level inferences is challenging for poorly defined sewersheds. However, members of our team have long-standing collaborations with the City of Atlanta and Fulton County and have worked closely with them to refine metro Atlanta sewer maps (20), which increases confidence in our results. Finally, combined sewer and sanitary sewer overflows are common in cities with older sewerage infrastructure, like Atlanta, which could affect pathogen concentrations observed in influent. Several reportable events occurred in the days prior to our sampling dates but most were < 100 gallons and likely had no discernable effect.

There were additional limitations to this study. First, we only tested wastewater at three timepoints (across two seasons). Substantial variability in AR Enterobacterales concentrations between winter and spring sampling rounds suggests that additional sampling might have allowed us to measure relationships with sewershed-level sociodemographic characteristics with less uncertainty. A recent Swiss study estimated that sampling at least once per month results in a 95% CI width below ± 0.5% (58). Second, we did not specifically account for recent rainfall or surface temperature in our statistical analyses although we did account for sampling round in all statistical analyses in an effort to account for differing meteorological conditions across timepoints. Recent studies have found mixed impacts of recent rainfall and temperature on AR Enterobacterales concentrations in wastewater (7, 58–60). Third, we were unable to consider physical differences in sewer systems, including conveyance time, piping material, conductivity, or the presence of biofilms, that could influence replication of AR Enterobacterales, their persistence and viability in sewers, or their ability to exchange ARGs (59). These features could have theoretically influenced differences in AR Enterobacterales concentrations in wastewater although given that sewershed population size (approximated in our study by flow rate) is a major predictor of ARG and AR bacteria concentrations in wastewater (55, 61), inputs from community and healthcare settings likely exert greater influence on AR Enterobacterales concentrations in untreated wastewater than sewer infrastructure. Fourth, recent, sewershed-level estimates of septic system reliance in metro Atlanta are not publicly available. Fifth, we used selective media to identify presumptive *E. coli* and *K. pneumoniae*, and it was not feasible to conduct species confirmation on every colony we counted. However, among the random subset of FQ-R Enterobacterales, 3GC-R *E. coli*, and 3GC-R KEC colonies that we did screen, we confirmed that the majority were species of interest, increasing confidence in our results. Finally, because we conducted analyses aggregated at the population level, our results are subject to ecological fallacy and should not be assumed to apply at the individual level. While identifying area-level characteristics that are associated with differences in AR burden across communities can provide helpful insights for population health, individual-level analyses are needed to determine if the associations between language and living conditions that we identified here also occur at the individual level.

### Conclusion

Wastewater surveillance could be a low-cost strategy for identifying community-level differences in AR Enterobacterales across urban centers in the United States. Understanding how accurately the sewershed-level differences we observed mirror trends in the human population will require robust comparisons with human colonization and infection data. Given major gaps in antibiotic resistance surveillance in the United States, wastewater surveillance merits further investigation as a novel tool for improving public health.

## Data Availability

Raw data needed to replicate our main analyses are available through the Open Science Framework (OSF) at https://osf.io/8fcj6/ and in Digital PCR results in the supplemental material.

## SUPPLEMENTAL MATERIAL

Supplemental material aem.01684-25-s0001.docx Supplemental methods, Fig. S1 to S4, and Tables S1 to S12.

Supplemental data aem.01684-25-s0002.xlsx Digital PCR results.

ASM does not own the copyrights to Supplemental Material that may be linked to, or accessed through, an article. The authors have granted ASM a non-exclusive, world-wide license to publish the Supplemental Material files. Please contact the corresponding author directly for reuse.
