# Supplementary material for: Using wastewater surveillance to investigate community-level differences in antibacterial resistance in a major urban center, USA
Source: Appl Environ Microbiol. 2025 Oct 30;91(11):e01684-25. doi: 10.1128/aem.01684-25 (PMC12628767; doi:10.1128/aem.01684-25)
Supplement: Supplemental material — Supplemental methods, Fig. S1 to S4, and Tables S1 to S12. [file aem.01684-25-s0001.docx]

**Using wastewater surveillance to investigate community-level differences in antibacterial resistance in a major urban center, USA**

**Supplemental File 1**

Cameron Goetgeluck, Oluwatosin Olojo, Stephen P. Hilton, Orlando Sablon III, Lorenzo Freeman, Patrick Person, David Clark, Robert H. Lyles, Caroline Sheikhzadeh, Marlene K. Wolfe, Maya L. Nadimpalli

**Appendix**

| **List of Figures** | **Page** |
| --- | --- |
| Figure S1: Number of staffed hospital beds and certified long term care facility (LTCF) beds per sewershed. | 4 |
| Figure S2: Metagenomic classification of all colonies growing on a mSuperCarba plate inoculated with undiluted, raw wastewater collected from the Johns Creek wastewater treatment plant, May 2023. | 5 |
| Figure S3: Flow-normalized concentrations of A) AR Enterobacterales and B) ARGs by sampling round, among 34 wastewater samples from 12 metro Atlanta sewersheds, 2022-2023. | 6 |
| Figure S4: Distributions of *bla*_CTX-M-1_ and *bla*_CTX-M-9_ group alleles among *E. coli* and *K. pneumoniae* recovered from Atlanta municipal wastewater. | 7 |
|  |  |

| **List of Tables** |  |
| --- | --- |
| Table S1: Proportion of suspected *Escherichia coli* or *Klebsiella*, *Enterobacter*, or *Citrobacter* spp. (KEC) detected in Atlanta wastewater confirmed to be *E. coli* or *K. pneumoniae* by endpoint PCR. | 8 |
| Table S2: Proportion of incident carbapenem-resistant Enterobacterales (CRE) infections harboring a carbapenemase gene in the metro Atlanta area, 2012-2022. | 9 |
| Table S3: Primer and probe sequences used for detection of antibiotic resistance genes in Atlanta wastewater using digital PCR. | 11 |
| Table S4: ACS5 variables used to tabulate sewershed-level sociodemographic characteristics of interest. | 11-13 |
| Table S5: Flow rates for each wastewater treatment plant (WWTP) and influent line sampled in this study. | 14 |
| Table S6: Associations between flow-normalized concentrations of third-generation cephalosporin-resistant (3GC) *E. coli*, 3GC-R KEC, and fluoroquinolone-resistant (FQ-R) Enterobacterales in Atlanta wastewater and number of staffed hospital beds and the number of certified long term care facility (LTCF) beds in a sewershed, 2022-2023. | 15 |
| Table S7: Detection of AR Enterobacterales in Atlanta area wastewater. | 16 |
| Table S8: Detection of *bla*_CTX-M-_group 1 and *bla*_KPC_ genes in Atlanta area wastewater. | 16 |
| Table S9: Distributions of *bla*_CTX-M_ group alleles among a random subset of *Escherichia coli* (n=41) and *Klebsiella pneumoniae* (n=24) detected in influent wastewater from 12 metro Atlanta sewersheds, 2022-2023. | 17 |
| Table S10: Statistically significant associations between concentrations of third generation cephalosporin-resistant (3GC-R) *E. coli*, 3GC-R KEC, and fluoroquinolone-resistant (FQ-R) Enterobacterales (not normalized by flow rate) in Atlanta wastewater and sewershed-level sociodemographic characteristics, 2022-2023. | 18 |
| Table S11: Associations between flow-normalized concentrations of third generation cephalosporin-resistant (3GC-R) *E. coli*, 3GC-R KEC, and fluoroquinolone-resistant (FQ-R) Enterobacterales in Atlanta wastewater and sewershed-level sociodemographic characteristics, 2022-2023. | 19-20 |
| Table S12: Sewershed-level sociodemographic characteristics that were strongly correlated, i.e., correlation co-efficient >\|0.7\|. | 21-22 |

**Supplementary Methods**

Given that no pink colonies on mSuperCarba were *E. coli* and few blue colonies were *K. pneumoniae*, we performed shotgun metagenomic sequencing of all MSuperCarba plate growth from one wastewater sample to gain insight into the diversity of phenotypically carbapenem-resistant bacteria present in Atlanta-area wastewater.

Briefly, 100μl of undiluted influent wastewater collected from the Johns Creek WWTP during the May 2023 sampling round was spread-plated on mSuperCarba. After overnight incubation at 37°C, all colonies were collected by adding 750μl of tryptic soy broth + 20% w/v glycerol to the plate, then gently scraping with a sterile L-shaped spreader to remove all colonies from the agar surface. This liquid ‘plate sweep’ was collected, and the process was repeated a second time to ensure that all colonies were removed from the plate. The resulting 1.5 mL liquid plate sweep was archived at -80°C until DNA extraction. Prior to DNA extraction, the sample was thawed and pelleted by centrifugation. The supernatant was discarded, the pellet was rinsed with PBS, and total DNA was extracted using Qiagen’s DNeasy PowerSoil Pro Kit (Germantown, MD).

The resulting DNA extract were sent to Omega Bioservices (Norcross, GA) for short-read, paired-end 150 bp sequencing on an Illumina Novaseq 6000 System; approximately 8 Gb of data was generated. Fastq sequences were filtered to remove sequencing adaptors and low quality reads using FastQC.^1^ We used the reduced 500GB NCBI RefSeq taxonomic database^2^ to conduct taxonomic profiling using Kraken2^3^ and used Bracken (Bayesian Re-estimation of Abundance after Classification with Kraken)^4^ to estimate relative abundance of classified bacteria. We visualized taxonomic classification results using Pavian.^5^

References

1.	Andrews S. s-andrews/FastQC [Internet]. 2024 [cited 2024 Sep 25]. Available from: https://github.com/s-andrews/FastQC

2.	Wright RJ, Comeau AM, Langille MGI. From defaults to databases: parameter and database choice dramatically impact the performance of metagenomic taxonomic classification tools. Microb Genom. 2023 Mar;9(3):000949. PMCID: PMC10132073

3.	Wood DE, Lu J, Langmead B. Improved metagenomic analysis with Kraken 2. Genome Biol. 2019 28;20(1):257. PMCID: PMC6883579

4.	Lu J, Breitwieser FP, Thielen P, Salzberg SL. Bracken: estimating species abundance in metagenomics data. PeerJ Comput Sci. PeerJ Inc.; 2017 Jan 2;3:e104.

5.	Pavian: interactive analysis of metagenomics data for microbiome studies and pathogen identification | Bioinformatics | Oxford Academic [Internet]. [cited 2024 Sep 25]. Available from: https://academic.oup.com/bioinformatics/article/36/4/1303/5573755

6.	Foxman B, Salzman E, Gesierich C, Gardner S, Ammerman M, Eisenberg M, Wigginton K. Wastewater surveillance of antibiotic resistant bacteria for public health action: Potential and Challenges. medRxiv. 2024 Jan 1;2024.03.31.24305136.

**Figures**

**Figure S1.** Number of staffed hospital beds and certified long term care facility (LTCF) beds per sewershed.

*Note*: High confidence assignments were squarely within the boundaries of a given sewershed; medium or low confidence assignments were relatively close to sewershed boundaries**.** Any sewershed that is not depicted was found to have no hospital nor LTCF beds.

**Figure S2.** Metagenomic classification of all colonies growing on a mSuperCarba plate inoculated with undiluted, raw wastewater collected from the Johns Creek wastewater treatment plant, May 2023.

**Figure S3.** Flow-normalized concentrations of **A)** AR Enterobacterales and **B)** ARGs by sampling round, among 34 wastewater samples from 12 metro Atlanta sewersheds, 2022-2023.

**Figure S4.** Distributions of *bla*_CTX-M-1_ and *bla*_CTX-M-9_ group alleles among *E. coli* and *K. pneumoniae* recovered from Atlanta municipal wastewater.

*Note:* We selected a random subset of up to 3 *bla*_CTX-M_-harboring *E. coli* and 3 *K. pneumoniae* per sewershed, per round, for sequencing. We sequenced the forward strand of the *bla*_CTX-M_ amplicon using Sanger sequencing and determined whether a *bla*_CTX-M_ allele belonging to group 1, 2, 8, 9, 25, 64, 151, or 137 was harbored using BLASTn. We used a 98% identity threshold to identify matches. A total of 65 isolates were sequenced. Atlanta sewershed shapefiles were obtained from the Atlanta Department of Watershed Management, and any areas outside city boundaries were estimated by digitizing maps from an online report (Dekalb County, 2022). Shapefiles for Fulton County were obtained from the Fulton County Department of Water Services. Background map data source: © OpenStreetMap contributors. Map prepared using QGIS 3.32 (QGIS Development Team, 2023).

**Tables**

**Table S1**. Proportion of suspected *Escherichia coli* or *Klebsiella*, *Enterobacter*, or *Citrobacter* spp. (KEC) detected in Atlanta wastewater confirmed to be *E. coli* or *K. pneumoniae* by endpoint PCR.

| Culture Media | Colony Phenotype | Number of colonies screened | Confirmed *E. coli*  n (%) | Confirmed *K. pneumoniae*  n (%) |
| --- | --- | --- | --- | --- |
| Chromagar ESBL | Pink (presumptive *E. coli*) | 84 | 61 (73) | -- |
| Chromagar ESBL | Blue (presumptive KEC) | 115 | -- | 27 (23) |
| MacConkey + 4 mg/L ciprofloxacin | Pink or tan (presumptive *E. coli*) | 149 | 103 (69) |  |
| MSuperCarba^a^ | Pink (presumptive *E. coli*) | 5 | 0 | -- |
| MSuperCarba^a^ | Blue (presumptive KEC) | 22 | -- | 1 (5) |

^a^Given the low proportion of presumptive *E. coli* and KEC that were confirmed to be the species of interest, quantitative findings from MSuperCarba are not presented in this manuscript.

*Note*: We were unable to regrow a substantial portion of presumptive carbapenem-resistant *E. coli* or KEC that we had archived on antibiotic-supplemented media for species confirmation, a challenge that other U.S. labs have noted.^6^

**Table S2**. Proportion of incident carbapenem-resistant Enterobacterales (CRE) infections harboring a carbapenemase gene in the metro Atlanta area, 2012-2022.

| Year | 2012 | 2013 | 2014 | 2015 | 2016 | 2017 | 2018 | 2019 | 2020 | 2021 | 2022 |
| --- | --- | --- | --- | --- | --- | --- | --- | --- | --- | --- | --- |
| % CRE harboring a carbapenemase gene | 9.7 | 20.0 | 19.4 | 25.7 | 8.5 | 9.4 | 22.9 | 16.3 | 20.5 | 24.0 | 23.1 |

*Note*: Data provided courtesy of the Georgia Emerging Infections Program.

**Table S3**. Primer and probe sequences used for detection of antibiotic resistance genes in Atlanta wastewater using digital PCR.

| Target gene |  | Sequence (5’ – 3’) | Amplicon size (bp) | Reference |
| --- | --- | --- | --- | --- |
| *bla*_CTX-M-_group 1 | F    R    Probe | ACCAACGATATCGCGGTGAT    ACATCGCGACGGCTTTCT    6-FAM-TCGTGCGCCGCTG-MGB-NFQ | 101 | Colomer-Lluch et al. (2011a) |
| *bla*_KPC_ | F    R    Probe | GACGGAAAGCTTACAAAAACTGAC    CTTGTCATCCTTGTTAGGCG    FAM-ACTGGGCAGTCGGAGACAAAACCGGA-BHQ1 | 259 | Oliveira et al. (2020)    Poirel et al. (2011)    Oliveira et al. (2020) |
| 16S rRNA | F    R    Probe | GAATGCCACGGTGAATACGTT    TCCCTACGGTTACCTTGTTACG    FAM-CACACCGCCCGTCACACCATGGGAG-BHQ1 | 157 | Oliveira et al. (2020) |

**Table S4.** ACS5 variables used to tabulate sewershed-level sociodemographic characteristics of interest.

| **Variable** | **Numerator** | **Description** | **Denominator** | **Description** |
| --- | --- | --- | --- | --- |
| % Speaks language other than English at home | B99162_003 | Total speak languages other than English for the population 5 years and over | B99162_001 | Total population 5 years and over |
| % Public health insurance | B27010_007 | Total under 19 years of age with Medicaid/means-tested public coverage only | B01003_001 | Total population |
|  | B27010_023 | Total 19-34 years of age with Medicaid/means-tested public coverage only |  |  |
|  | B27010_039 | Total 35-64 years of age with Medicaid/means-tested public coverage only |  |  |
| % No health insurance | B27010_017 | Total under 19 years of age with no health insurance | B01003_001 | Total population |
|  | B27010_033 | Total 19-34 years of age with no health insurance |  |  |
|  | B27010_050 | Total 35-64 years of age with no health insurance |  |  |
|  | B27010_066 | Total 65 years of age and over with no health insurance |  |  |
| Median household income | B19013_001 | Median household income in the past 12 months (2020 inflation-adjusted dollars) |  |  |
| % living in poverty | B29003_002 | Income in the past 12 months below the federal poverty line | B29003_001 | Citizen, Voting-Age population by poverty status |
| % living in crowded households | B25014_005 | Owner Occupied household with 1.01 to 1.50 occupants per room | B25014_001 | Total households by occupants per room |
|  | B25014_006 | Owner Occupied household with 1.51 to 2.00 occupants per room |  |  |
|  | B25014_007 | Owner Occupied household with 2.01 or more occupants per room |  |  |
|  | B25014_011 | Renter Occupied household with 1.01 to 1.50 occupants per room |  |  |
|  | B25014_012 | Renter Owner Occupied household with 1.51 to 2.00 occupants per room |  |  |
|  | B25014_013 | Renter Owner Occupied household with 2.01 or more occupants per room |  |  |
| % Hispanic | B03002_012 | Total Hispanic or Latino | B03002_001 | Total population |
| % Non-Hispanic White | B03002_003 | Total not Hispanic or Latino, White alone | B03002_001 | Total population |
| % Non-Hispanic Black | B03002_004 | Total not Hispanic or Latino, Black alone | B03002_001 | Total population |
| % Non-Hispanic Asian | B03002_006 | Total not Hispanic or Latino, Asian alone | B03002_001 | Total population |
| % completed at least high school^a^ | B15003_003 | Total population 25 years and older with highest level of education attainment being nursery school | B15003_001 | Total population 25 years and older |
|  | B15003_004 | Total population 25 years and older with highest level of education attainment being kindergarten |  |  |
|  | B15003_005 | Total population 25 years and older with highest level of education attainment being 1^st^ grade |  |  |
|  | B15003_006 | Total population 25 years and older with highest level of education attainment being 2^nd^ grade |  |  |
|  | B15003_007 | Total population 25 years and older with highest level of education attainment being 3^rd^ grade |  |  |
|  | B15003_008 | Total population 25 years and older with highest level of education attainment being 4^th^ grade |  |  |
|  | B15003_009 | Total population 25 years and older with highest level of education attainment being 5^th^ grade |  |  |
|  | B15003_010 | Total population 25 years and older with highest level of education attainment being 6^th^ grade |  |  |
|  | B15003_011 | Total population 25 years and older with highest level of education attainment being 7^th^ grade |  |  |
|  | B15003_012 | Total population 25 years and older with highest level of education attainment being 8^th^ grade |  |  |
|  | B15003_013 | Total population 25 years and older with highest level of education attainment being 9^th^ grade |  |  |
|  | B15003_014 | Total population 25 years and older with highest level of education attainment being 10^th^ grade |  |  |
|  | B15003_015 | Total population 25 years and older with highest level of education attainment being 11^th^ grade |  |  |
|  | B15003_016 | Total population 25 years and older with highest level of education attainment being 12^th^ grade, no diploma |  |  |

*Note*: Unless otherwise noted, all variables were tabulated as the sum of all numerator variables divided by the denominator variable.

^a^Tabulated as 1-(Sum of all numerator variables)/denominator variable.

**Table S5**. Flow rates for each wastewater treatment plant (WWTP) and influent line sampled in this study.

|  |  | Flow Rate (MGD) | | |
| --- | --- | --- | --- | --- |
| Location | Type | Dec 2022 | March 2023 | May 2023 |
| South River | Treatment Plant | 17.49 | 35.4 | 23.64 |
| Utoy Creek | Treatment Plant | 29.0 | 54.0 | 30.0 |
| RM Clayton | Treatment Plant | 91.13 | 133.60 | 86.27 |
| Big Creek | Treatment Plant | 18.41 | 26.15 | 16.91 |
| Camp Creek | Treatment Plant | 19.23 | 21.5 | 18.47 |
| Johns Creek | Treatment Plant | 9.44 | 11.6 | 9.02 |
| Little River | Treatment Plant | 0.91 | 1.09 | 0.84 |
| Peachtree Creek | Influent Line | 57.3 | | |
| Nancy Creek | Influent Line | 14.8 | | |
| Flint | Influent Line | 3.2 | | |
| Jonesboro | Influent Line | 1 | | |
| Phillip Lee | Influent Line | 11 | | |

Note: MGD=Million gallons per day. Daily flow rates on the date(s) of sampling were available for WWTPs. Average annual flow rates were available for influent lines. All data were provided by the City of Atlanta and Fulton County.

**Table S6**. Associations between flow-normalized concentrations of third-generation cephalosporin-resistant (3GC) *E. coli*, 3GC-R KEC, and fluoroquinolone-resistant (FQ-R) Enterobacterales in Atlanta wastewater and number of staffed hospital beds and the number of certified long term care facility (LTCF) beds in a sewershed, 2022-2023.

| Sewershed-level characteristic | Outcome  (log10 CFU/day) | Estimate | Std. Error | Wald test statistic | Pr (>\|W\|) |
| --- | --- | --- | --- | --- | --- |
| No. of hospital beds | 3GC-R *E. coli* | 0.0007 | 8.72E-05 | 56.49 | 5.65E-14 |
|  | 3GC-R KEC | 0.0004 | 1.55E-04 | 6.33 | 1.18E-02 |
|  | FQ-R Enterobacterales | 0.0004 | 1.13E-04 | 14.38 | 1.50E-04 |
| No. of LTCF beds | 3GC-R *E. coli* | 0.0008 | 1.39E-04 | 32.32 | 1.30E-08 |
|  | 3GC-R KEC | 0.0005 | 2.45E-04 | 4.80 | 2.85E-02 |
|  | FQ-R Enterobacterales | 0.0006 | 1.51E-04 | 17.19 | 3.39E-05 |

**Table S7. Detection of AR Enterobacterales in Atlanta area wastewater.**

| Outcome assessed | No. of positive samples  n=34 (%) | Median log_10_ CFU per 100 mL [minimum, maximum] | Median log_10_CFU per day [minimum, maximum]^a^ |
| --- | --- | --- | --- |
| 3GC-R *E. coli* | 27 (79.4) | 4.47 [1.70, 5.82] | 15.31 [11.21, 17.20] |
| 3GC-R KEC | 29 (85.2) | 4.39 [1.70, 6.69] | 15.51 [11.21, 17.54] |
| FQ-R Enterobacterales | 32 (94.1) | 4.55 [2.70, 6.32] | 15.57 [12.21, 17.17] |

*Note*: 3GC-R=third-generation cephalosporin-resistant, FQ-R=fluoroquinolone-resistant, KEC=*Klebsiella* spp., *Enterobacter* spp., *Citrobacter* spp. Findings for carbapenem-resistant Enterobacterales are not depicted because of low confidence in results.

^a^Flow-normalized concentrations were calculated by multiplying bacterial concentrations (in CFU/L) by flow rate (in liters/day). For WWTPs, average daily flow rates were used. For influent lines, average annual flow rates were used.

**Table S8. Detection of *bla*_CTX-M_-group 1 and *bla*_KPC_ genes in Atlanta area wastewater.**

| Outcome assessed | No. of positive samples  n=34 (%) | Median concentrations normalized by 16S rRNA [minimum, maximum] | Median log_10_ concentrations normalized by 16S rRNA and flow rate [minimum, maximum]^a^ |
| --- | --- | --- | --- |
| *bla*_CTX-M_-group 1 | 34 (100) | 0.001 [4.79 x 10^-6^, 0.012] | 4.86 [1.22, 6.43] |
| *bla*_KPC_ | 34 (100) | 0.002 [1.04 x 10^-4^, 0.047] | 5.00 [3.13, 7.01] |

^a^For WWTPs, average daily flow rates were used. For influent lines, average annual flow rates were used.

**Table S9.** Distributions of *bla*_CTX-M_ group alleles among a random subset of *Escherichia coli* (n=41) and *Klebsiella pneumoniae* (n=24) detected in influent wastewater from 12 metro Atlanta sewersheds, 2022-2023.

|  | *Escherichia coli* (n=41) | | *Klebsiella pneumoniae* (n=24) | |
| --- | --- | --- | --- | --- |
|  | bla_CTX-M-1_ | bla_CTX-M-9_ | bla_CTX-M-1_ | bla_CTX-M-9_ |
| Big Creek | 5 | 1 | 3 | 0 |
| Camp Creek | 3 | 0 | 3 | 0 |
| Johns Creek | 5 | 0 | 3 | 0 |
| Little River | 3 | 2 | 1 | 0 |
| RM Clayton | 1 | 0 | 3 | 0 |
| South River | 3 | 1 | 1 | 0 |
| Utoy Creek | 0 | 1 | 0 | 0 |
| Nancy Creek^a^ | 4 | 0 | 2 | 0 |
| Peachtree Creek^a^ | 2 | 2 | 2 | 0 |
| Flint^b^ | 3 | 0 | 2 | 0 |
| Jonesboro^b^ | 3 | 0 | 3 | 0 |
| Phillip Lee^c^ | 2 | 0 | 1 | 0 |

^a^Influent lines of RM Clayton WWTP

^b^Influent lines of SR WWTP

^c^Influent lines of Utoy Creek WWTP

**Table S10.** Statistically significant associations between concentrations of third generation cephalosporin-resistant (3GC-R) *E. coli*, 3GC-R KEC, and fluoroquinolone-resistant (FQ-R) Enterobacterales (not normalized by flow rate) in Atlanta wastewater and sewershed-level sociodemographic characteristics, 2022-2023.

| Sewershed-level characteristic | Outcome (log10 CFU/L) | Estimate | Std. Error | Wald test statistic | Pr (>\|W\|) |
| --- | --- | --- | --- | --- | --- |
| % living in crowded households^a^ | FQ-R Enterobacterales | 0.35 | 0.14 | 5.88 | 1.53E-02 |
| % Hispanic^b^ | 3GC-R *E. coli* | 0.10 | 0.03 | 12.64 | 3.78E-04 |
|  | 3GC-R KEC | 0.15 | 0.04 | 15.74 | 7.26E-05 |
| % Non-Hispanic Asian^c^ | 3GC-R *E. coli* | 0.05 | 0.01 | 22.30 | 2.33E-06 |
|  | 3GC-R KEC | 0.05 | 0.02 | 10.77 | 1.03E-03 |
| % Speaks language other than English at home^d^ | 3GC-R *E. coli* | 0.04 | 0.01 | 31.40 | 2.09E-08 |
|  | 3GC-R KEC | 0.04 | 0.01 | 20.44 | 6.14E-06 |

*Note*: Models accounted for repeated measures within each sewershed, sampling round, and number of hospital or long-term care facility beds within each sewershed. Statistical significance was defined as p<0.167.

^a^Defined as greater than 1.01 persons/room, not including kitchens or bathrooms. Tabulated as number of owner-occupied households with greater than 1.01 occupants/room divided by total households.

^b^Tabulated as number of persons self-identifying as Hispanic or Latino divided by the total population.

^c^Tabulated as number of persons self-identifying as non-Hispanic Asian alone divided by the total population.

^d^Tabulated as the number of persons 5 years and over who speak a language other than English at home divided by the total population. This variable was highly correlated with percent Hispanic or Latino (ρ=0.80) and percent non-Hispanic Asian living in a sewershed (ρ=0.95).

**Table S11**. Associations between flow-normalized concentrations of third generation cephalosporin-resistant (3GC-R) *E. coli*, 3GC-R KEC, and fluoroquinolone-resistant (FQ-R) Enterobacterales in Atlanta wastewater and sewershed-level sociodemographic characteristics, 2022-2023.

| Sewershed-level characteristic | Outcome (log10 CFU/day) | Estimate | Std. Error | Wald test statistic | Pr (>\|W\|) |
| --- | --- | --- | --- | --- | --- |
| % Speaks language other than English at home | 3GC-R E. coli | 0.05 | 0.01 | 34.30 | 4.73E-09 |
| % Hispanic | 3GC-R E. coli | 0.12 | 0.03 | 17.75 | 2.51E-05 |
| % living in crowded households | 3GC-R E. coli | 0.06 | 0.01 | 17.27 | 3.23E-05 |
| % Non-Hispanic Asian | 3GC-R E. coli | 0.50 | 0.12 | 16.88 | 3.98E-05 |
| % living in poverty | 3GC-R E. coli | -0.03 | 0.02 | 1.69 | 1.94E-01 |
| % Public health insurance | 3GC-R E. coli | -0.02 | 0.02 | 0.82 | 3.66E-01 |
| % completed at least high school | 3GC-R E. coli | 0.04 | 0.05 | 0.68 | 4.09E-01 |
| % No health insurance | 3GC-R E. coli | -0.01 | 0.01 | 0.61 | 4.33E-01 |
| % Non-Hispanic Black | 3GC-R E. coli | -0.04 | 0.05 | 0.57 | 4.50E-01 |
| Median household income | 3GC-R E. coli | 0.00 | 0.00 | 0.05 | 8.30E-01 |
| % Hispanic | 3GC-R KEC | 0.17 | 0.04 | 19.66 | 9.24E-06 |
| % Speaks language other than English at home | 3GC-R KEC | 0.05 | 0.01 | 15.83 | 6.91E-05 |
| % living in crowded households | 3GC-R KEC | 0.61 | 0.19 | 10.24 | 1.38E-03 |
| % Non-Hispanic Asian | 3GC-R KEC | 0.06 | 0.02 | 8.51 | 3.53E-03 |
| % living in poverty | 3GC-R KEC | -0.05 | 0.03 | 1.84 | 1.75E-01 |
| % Public health insurance | 3GC-R KEC | -0.03 | 0.03 | 0.86 | 3.54E-01 |
| % No health insurance | 3GC-R KEC | -0.05 | 0.07 | 0.56 | 4.54E-01 |
| % Non-Hispanic Black | 3GC-R KEC | -0.01 | 0.01 | 0.54 | 4.64E-01 |
| % completed at least high school | 3GC-R KEC | 0.04 | 0.07 | 0.44 | 5.08E-01 |
| Median household income | 3GC-R KEC | 0.00 | 0.00 | 0.01 | 9.32E-01 |
| % living in crowded households | FQ-R Enterobacterales | 0.70 | 0.14 | 26.43 | 2.73E-07 |
| % Speaks language other than English at home | FQ-R Enterobacterales | 0.03 | 0.02 | 3.65 | 5.62E-02 |
| % Non-Hispanic Asian | FQ-R Enterobacterales | 0.04 | 0.02 | 2.83 | 9.23E-02 |
| % Hispanic | FQ-R Enterobacterales | 0.00 | 0.00 | 0.36 | 5.47E-01 |
| Median household income | FQ-R Enterobacterales | -0.02 | 0.03 | 0.30 | 5.83E-01 |
| % living in poverty | FQ-R Enterobacterales | 0.02 | 0.04 | 0.22 | 6.39E-01 |
| % No health insurance | FQ-R Enterobacterales | -0.02 | 0.06 | 0.10 | 7.49E-01 |
| % Non-Hispanic Black | FQ-R Enterobacterales | 0.00 | 0.01 | 0.07 | 7.96E-01 |
| % completed at least high school | FQ-R Enterobacterales | 0.00 | 0.03 | 0.00 | 9.74E-01 |
| % Public health insurance | FQ-R Enterobacterales | 0.00 | 0.05 | 0.00 | 9.83E-01 |

*Note*: Models accounted for repeated measures within each sewershed, sampling round, and number of hospital or long-term care facility beds within each sewershed.

**Table S12**. Sewershed-level sociodemographic characteristics that were strongly correlated, i.e., correlation co-efficient >|0.7|.

| Variable 1 | Variable 2 | Correlation Coefficient |
| --- | --- | --- |
| At least high school % | Median Household Income | 0.917 |
| At least high school % | non hispanic white % | 0.859 |
| At least high school % | non hispanic black % | -0.853 |
| At least high school % | uninsured % | -0.898 |
| At least high school % | poverty % | -0.908 |
| At least high school % | public ins % | -0.934 |
| Median Household Income | non hispanic white % | 0.933 |
| Median Household Income | At least high school % | 0.917 |
| Median Household Income | poverty % | -0.833 |
| Median Household Income | uninsured % | -0.892 |
| Median Household Income | non hispanic black % | -0.901 |
| Median Household Income | public ins % | -0.911 |
| hispanic % | speak other lang % | 0.796 |
| hispanic % | non hispanic white % | 0.727 |
| hispanic % | public ins % | -0.718 |
| hispanic % | non hispanic black % | -0.787 |
| non hispanic asian % | speak other lang % | 0.949 |
| non hispanic asian % | public ins % | -0.725 |
| non hispanic asian % | non hispanic black % | -0.778 |
| non hispanic black % | public ins % | 0.949 |
| non hispanic black % | uninsured % | 0.862 |
| non hispanic black % | poverty % | 0.814 |
| non hispanic black % | non hispanic asian % | -0.778 |
| non hispanic black % | hispanic % | -0.787 |
| non hispanic black % | speak other lang % | -0.792 |
| non hispanic black % | At least high school % | -0.853 |
| non hispanic black % | Median Household Income | -0.901 |
| non hispanic black % | non hispanic white % | -0.984 |
| non hispanic white % | Median Household Income | 0.933 |
| non hispanic white % | At least high school % | 0.859 |
| non hispanic white % | hispanic % | 0.727 |
| non hispanic white % | poverty % | -0.813 |
| non hispanic white % | uninsured % | -0.877 |
| non hispanic white % | public ins % | -0.943 |
| non hispanic white % | non hispanic black % | -0.984 |
| poverty % | uninsured % | 0.96 |
| poverty % | public ins % | 0.924 |
| poverty % | non hispanic black % | 0.814 |
| poverty % | non hispanic white % | -0.813 |
| poverty % | Median Household Income | -0.833 |
| poverty % | At least high school % | -0.908 |
| public ins % | non hispanic black % | 0.949 |
| public ins % | poverty % | 0.924 |
| public ins % | uninsured % | 0.918 |
| public ins % | hispanic % | -0.718 |
| public ins % | non hispanic asian % | -0.725 |
| public ins % | speak other lang % | -0.727 |
| public ins % | Median Household Income | -0.911 |
| public ins % | At least high school % | -0.934 |
| public ins % | non hispanic white % | -0.943 |
| speak other lang % | non hispanic asian % | 0.949 |
| speak other lang % | hispanic % | 0.796 |
| speak other lang % | public ins % | -0.727 |
| speak other lang % | non hispanic black % | -0.792 |
| uninsured % | poverty % | 0.96 |
| uninsured % | public ins % | 0.918 |
| uninsured % | non hispanic black % | 0.862 |
| uninsured % | non hispanic white % | -0.877 |
| uninsured % | Median Household Income | -0.892 |
| uninsured % | At least high school % | -0.898 |

Correlation coefficients generated using the corr() function in R. Variables were created using ACS5 variables described in **Table S4**. Only correlations with correlation co-efficient >0.7 are depicted.
